# Antibacterial Compounds Isolated from Endophytic Fungi Reported from 2021 to 2024

**DOI:** 10.3390/antibiotics14070644

**Published:** 2025-06-25

**Authors:** Humberto E. Ortega, Daniel Torres-Mendoza, Luis Cubilla-Rios

**Affiliations:** 1 Departamento de Química Orgánica, Facultad de Ciencias Naturales, Exactas y Tecnología, Universidad de Panamá, Panamá 0824, Panama; humberto.ortegad@up.ac.pa (H.E.O.); daniel-t.torres-m@up.ac.pa (D.T.-M.); 2 Laboratorio de Bioorgánica Tropical, Facultad de Ciencias Naturales, Exactas y Tecnología, Universidad de Panamá, Panamá 0824, Panama; 3 Sistema Nacional de Investigación (SNI), Secretaría Nacional de Ciencia, Tecnología e Innovación, Ciudad del Saber, Clayton, Panamá 0816, Panama; 4 Vicerrectoría de Investigación y Postgrado, Universidad de Panamá, Panamá 0824, Panama

**Keywords:** endophytic fungi, secondary metabolites, antibacterial compounds, antimicrobial resistance

## Abstract

Plant endophytic fungi remain a significant source of novel bioactive compounds with uncommon structures rarely found in nature. The discovery of new antibiotics is crucial for combating the growing resistance of pathogenic bacteria, which poses a significant threat to global health. In this review, we examined 132 antibacterial compounds produced by endophytic fungi, reported between January 2021 and December 2024. The most frequently cited fungal genera were *Aspergillus* and *Penicillium*, with medicinal plants serving as the primary source of these fungi. Rice was the most used culture medium. A subset of the compounds exhibited biological activity comparable to that of clinically used antibiotics. Some of these molecules may serve as scaffolds for the development of more potent derivatives or synergy studies with antibiotics of medical relevance.

## 1. Introduction

**Figure 1:**
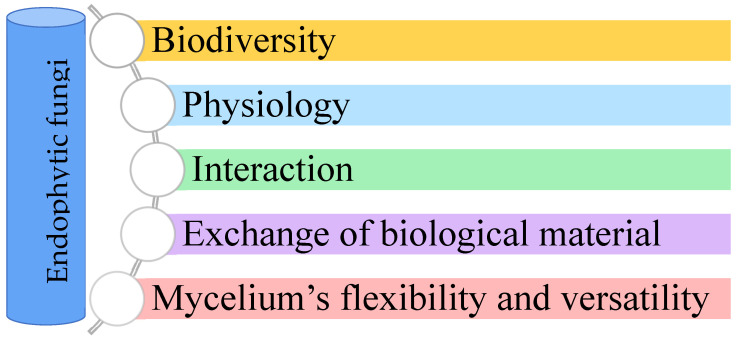
Elements that explain the capabilities of fungi to produce secondary metabolites.

Fungi were classified as part of the plant kingdom for a long period of time; it was not until 1959 that ecologist Robert Whittaker acknowledged them as a separate life form (or domain) [1]. Since then, a couple of elements have been added to the definition and understanding of this kingdom [2]. Understanding the capability of these organisms to synthesize a diverse range of secondary metabolites, including some with rare structural features and exceptional bioactivities, requires the consideration of several key elements (Figure 1): (1) **fungal biodiversity**―different species and strains of endophytic fungi can synthetize unique compounds with potential pharmacological, agricultural, industrial, or biotechnological applications [3,4,5,6]; (2) **fungal physiology**―the production of these metabolites is directly related to the physiology of the fungus, including its primary metabolism, genetic regulation, and response to stress and environmental conditions [7,8]; (3) **fungal interactions**―the interactions between fungi and their hosts, as well as those with other microorganisms, can induce or modulate the production of metabolites; these interactions can be mutualistic, symbiotic, or competitive, which directly impacts the biosynthesis of the molecules [9,10,11,12,13,14,15]; (4) **exchange of biological material horizontally/vertically**―the transfer of genetic material between fungi and their host or between fungi themselves can influence the production of metabolites, allowing the acquisition of genes that encode bioactive molecules [16,17,18,19,20]; (5) **mycelium’s flexibility and versatility**―the adaptation of the mycelium to different environmental conditions and substrates can affect the production of metabolites [21,22]; this is particularly relevant given that each diminutive fragment of the mycelium retains the inherent characteristics of its respective species or strain [22,23]. However, since these properties are intrinsically linked to the organism’s physiology, it is essential to emphasize their importance.

In addition to these factors influencing the production of secondary metabolites by endophytic fungi, an alternative pathway may be considered that could facilitate their application in synthetic biology [24,25,26,27,28]. This approach would enable the identification and characterization of biosynthetic routes for a metabolite with targeted applications.

Fungi have been the source of some of the most important drugs ever discovered, playing essential roles in the treatment of chronic infections, autoimmune diseases, and hypercholesterolemia. Among the most significant approved antibiotic drugs of fungal origin are penicillin G, penicillin V, cephalosporine C, fusidic acid, and pleuromutilin [29].

The urgent need for the discovery of new antibacterial molecules is well established, driven by the widespread use of existing antibiotics in human medicine, veterinary applications, and agriculture. This issue has become increasingly critical during and after the COVID-19 pandemic. Bacterial antimicrobial resistance (AMR) and infections due to antibiotic-resistant (ABR) pathogens were linked to approximately 5 million deaths in 2019, with 1.27 million being directly attributable to drug-resistant infections. There are several actions to reduce or mitigate the impact of AMR [30,31,32,33,34]. Here is where endophytic fungi and their metabolites provide a way to improve the efficacy of treatments for infectious diseases and address the need for new antimicrobial compounds.

Within the scope of this review, the literature analyzed highlights several factors that contribute to the production and isolation of diverse compounds, particularly those with novel structures and/or significant antibiotic activity.

## 2. Results

The data analyzed showed that around 65% of the culture mediums used to ferment endophytic fungi were rice with distilled water or with another supplement such as salt, peptone, sucrose, and malt extract broth. In nearly 50% of cases, the cultivation period was 3 to 4 weeks under static conditions at 25 to 28 °C.

**Figure 2:**
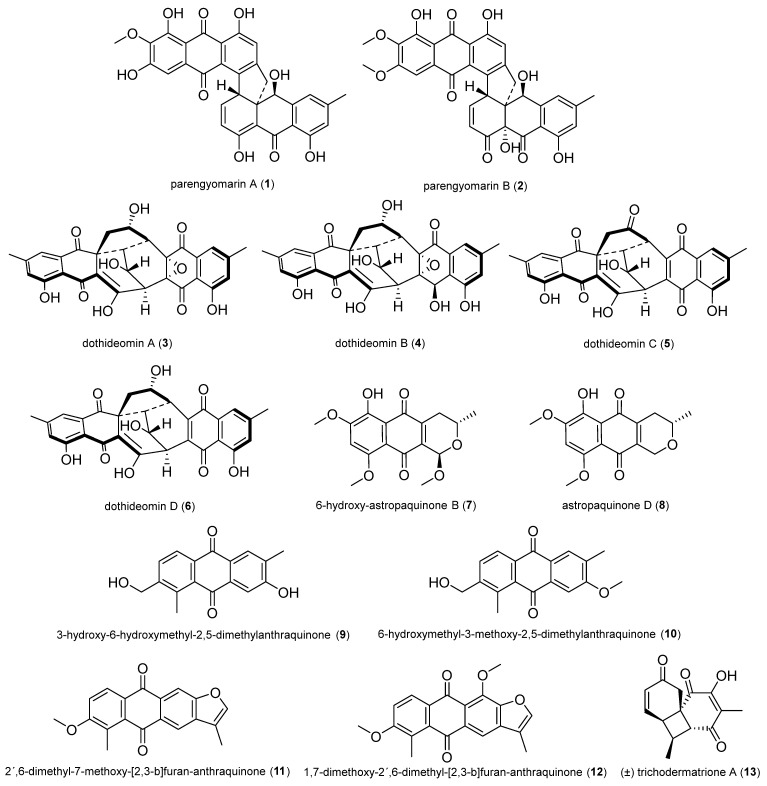
Quinones.

**Figure 3:**
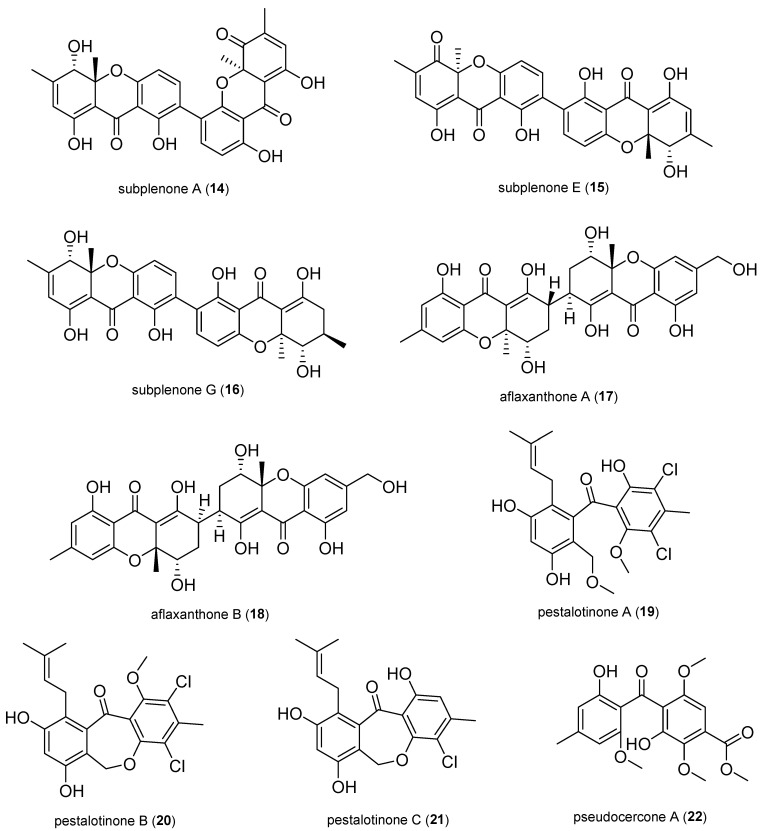
Xanthones and benzophenone derivatives.

**Figure 4:**
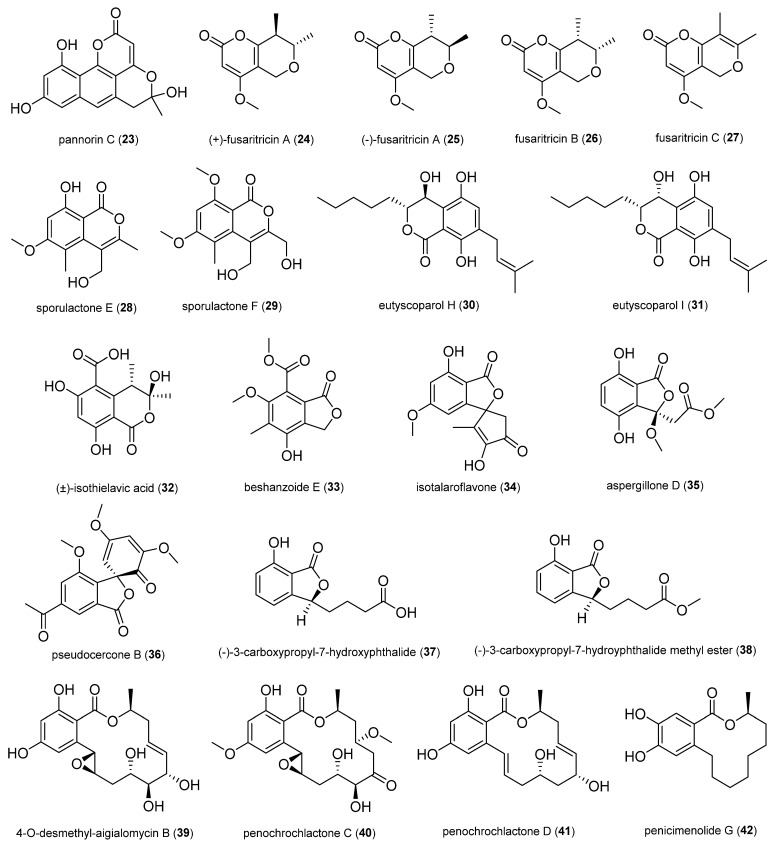
Pyrones and lactones.

**Figure 5:**
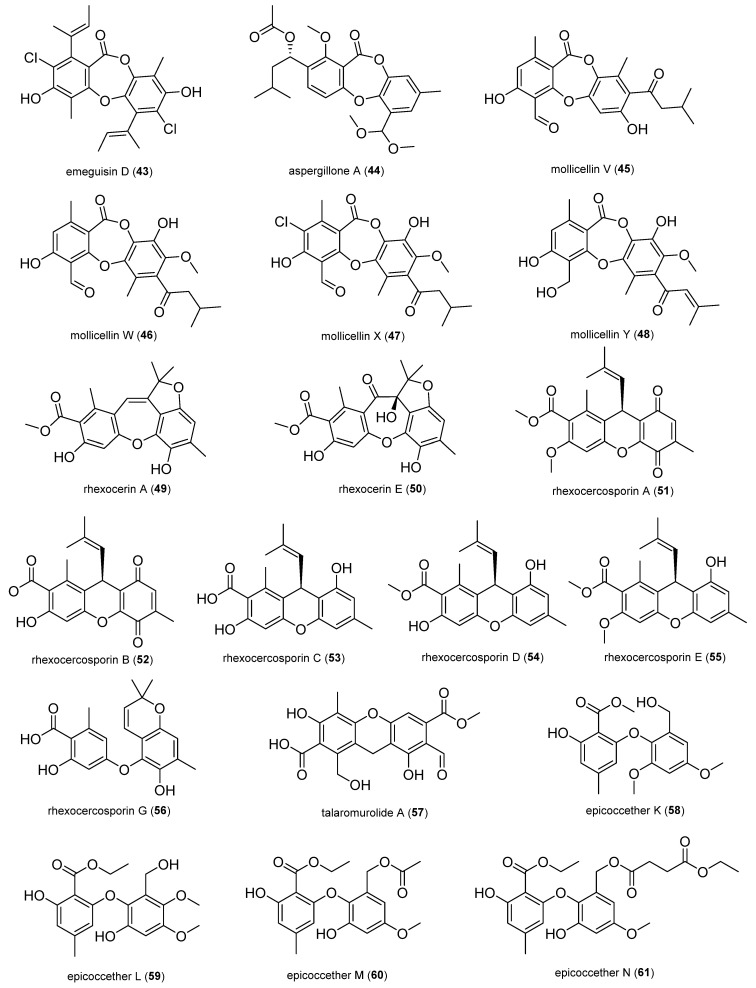
Depsidones and diphenyl ether derivatives.

**Figure 6:**
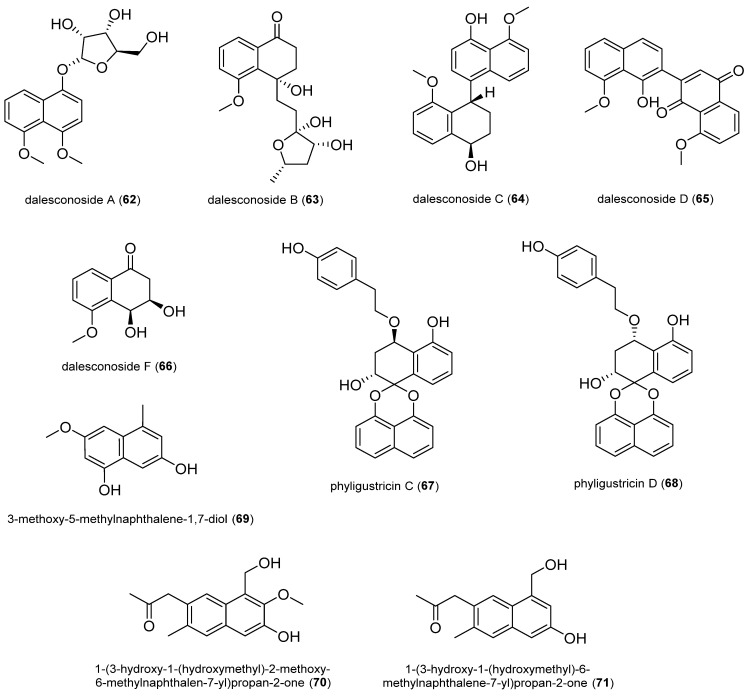
Naphthalene derivatives.

**Figure 7:**
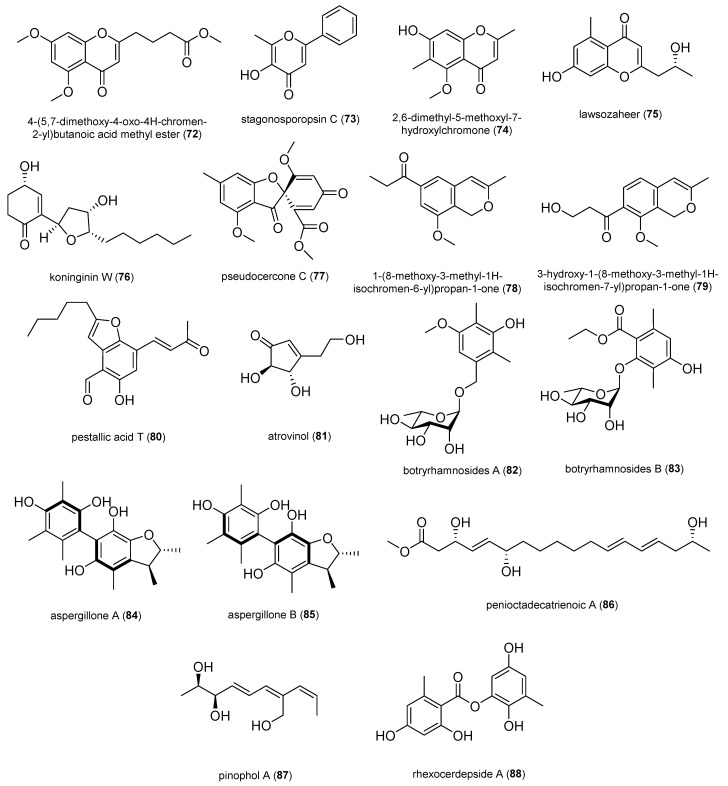
Other polyketides.

**Figure 8:**
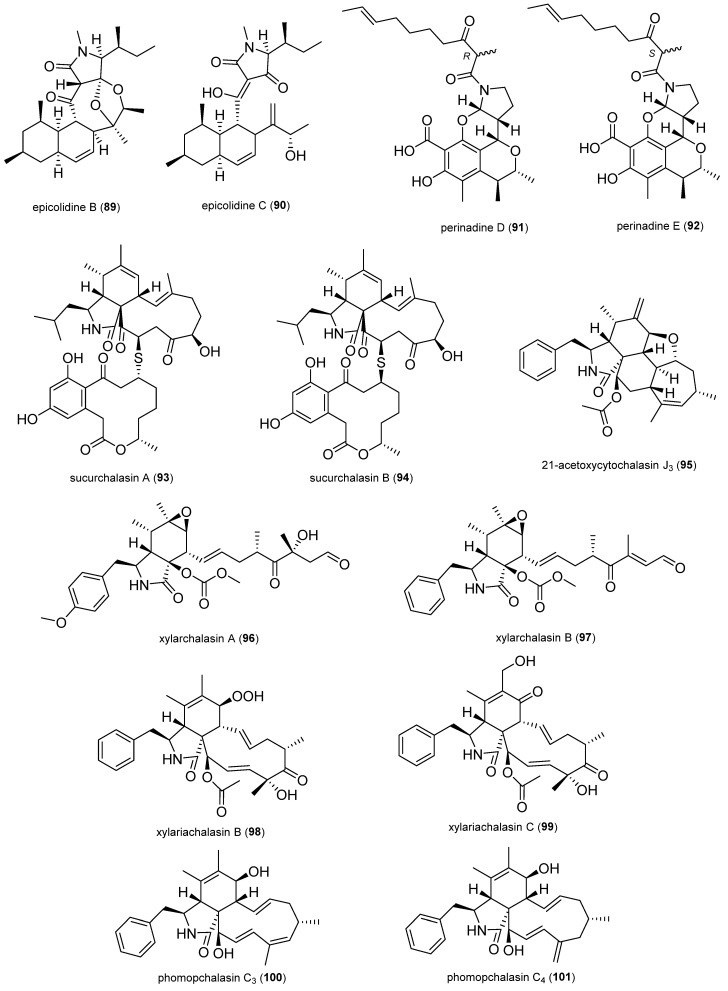
Nitrogen-containing compounds.

**Figure 9:**
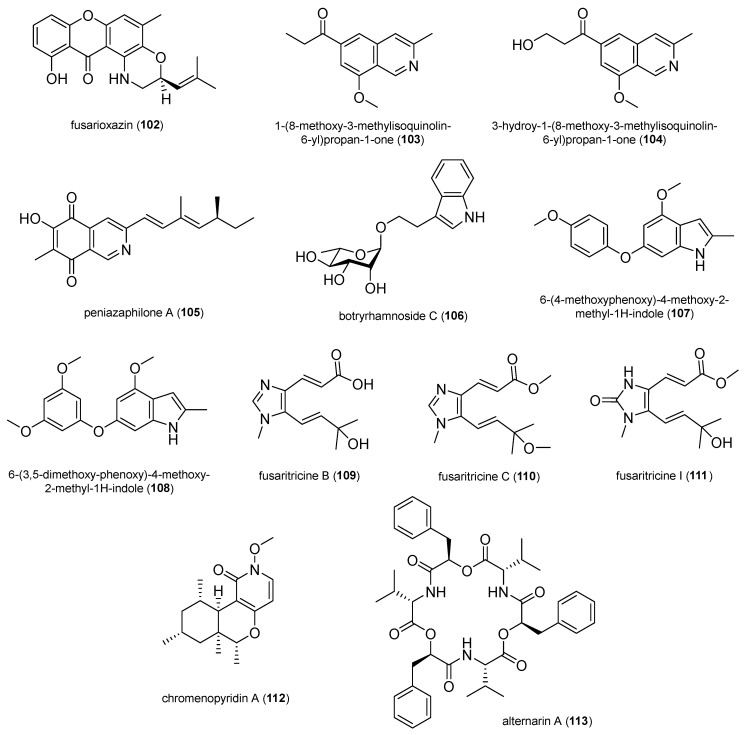
Nitrogen-containing compounds.

**Figure 10:**
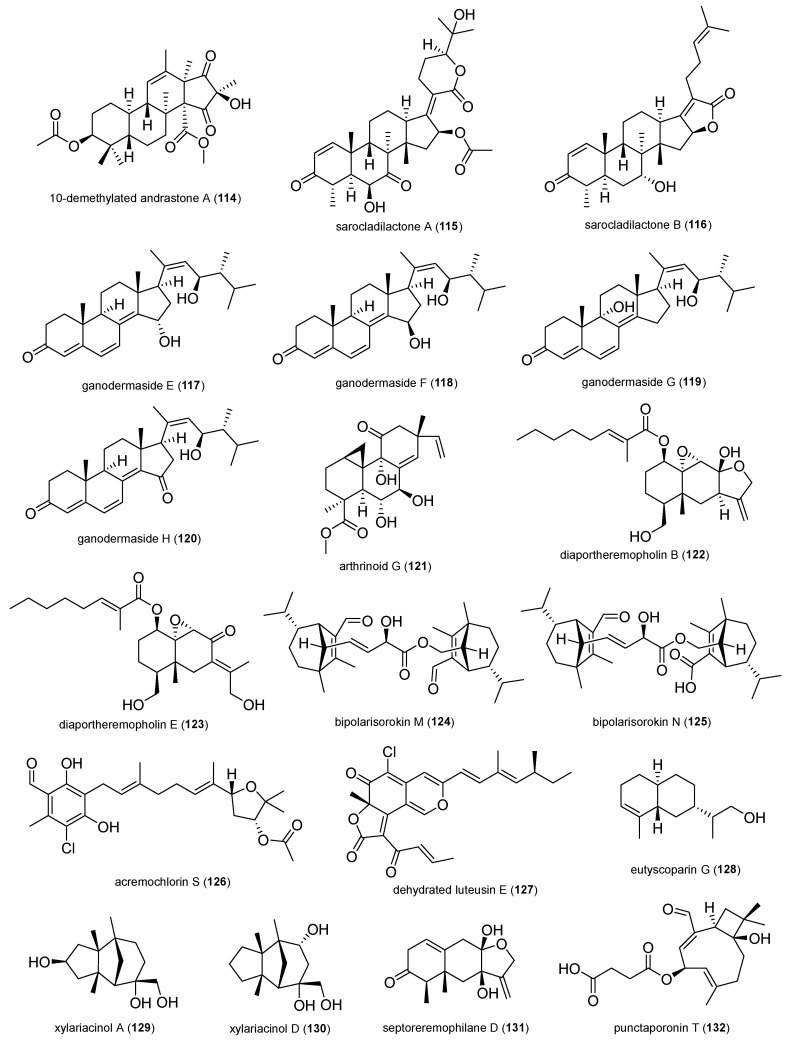
Terpenoids.

In total, 132 natural products were selected based on their structural skeleton and antibacterial activity. They were classified as polyketides (quinones, xanthones and benzophenone derivatives, pyrones and lactones, depsidones and diphenyl ether derivatives, naphthalene derivatives, and other polyketides), nitrogen-containing compounds, and terpenoids (Figure 2, Figure 3, Figure 4, Figure 5, Figure 6, Figure 7, Figure 8, Figure 9 and Figure 10). In Tables S1–S5, the antibacterial activity of the 132 compounds is compared with positive controls and separated according to the pathogenic bacteria used in the bioassays (*Escherichia coli*, *Pseudomonas* spp., *Bacillus* spp., *Staphylococcus aureus*, MRSA, and others); Table 1 shows some of the most active fungal metabolites compared to the antibiotic compound used as a positive control.

### 2.1. Polyketides

The term polyketide refers to a group of highly diverse secondary metabolites (enedyines, macrolides, polyenes, polyethers, and polyphenols) defined by their biosynthetic derivation through the condensation of acyl thioester precursors by polyketide synthase followed by further modification by the action of other enzymes. They exhibit a diverse spectrum of biological activities and serve as sources of pharmaceutical lead compounds. Examples of polyketides as antibiotics include erythromycin A, clarithromycin, azithromycin, and members of the tetracycline family [35].

#### 2.1.1. Quinones

Two new torrubielin derivatives, parengyomarin A (**1**) and parengyomarin B (**2**), were isolated from the endophytic fungus *Parengyodontium album* obtained from the mangrove *Avicennia marina*. Both compounds showed significant antibacterial activity against *S. aureus* and MRSA (MIC of 0.39 to 1.56 μM) [36].

Dothideomins A–D (**3**–**6**) are four new bisanthraquinones identified from *Dothideomycetes* sp. BMC-101, an endophytic fungus isolated from *Magnolia grandiflora* leaves. Their structures were characterized by an unusual 6/6/6/5/6/3/6/6 octocyclic scaffold (**3** and **4**) and a 6/6/6/5/6/6/6 heptacyclic scaffold (**5** and **6**), respectively. These compounds, especially **3** and **5**, exhibited potent antibacterial activity with MIC values ranging from 0.4 to 0.8 μg/mL [37].

6-hydroxy-astropaquinone B (**7**) and astropaquinone D (**8**) were isolated from *Fusarium napiforme* obtained from the mangrove *Rhizophora mucronate*. Both compounds exhibited moderate antibacterial activity against *S. aureus* NBRC13276 (MIC of 6.3 and 12.5 µg/mL, respectively) and *Pseudomonas aeruginosa* ATCC15442 (MIC of 6.3 µg/mL for both) [38].

3-hydroxy-6-hydroxymethyl-2,5-dimethylanthraquinone (**9**) and 6-hydroxymethyl-3-methoxy-2,5-dimethylanthraquinone (**10**) were isolated from *Phomopsis* sp. obtained from the root of *Nicotiana tabacum* L. Compounds **9** and **10** showed activity against MRSA ZR11, with an inhibition zone of 14.2 ± 2.0 and 14.8 ± 2.2 mm, respectively [39].

2′,6-dimethyl-7-methoxy-[2,3-b]furan-anthraquinone (**11**) and 1,7-dimethoxy-2′,6-dimethyl-[2,3-b]furan-anthraquinone (**12**) were isolated from *Aspergillus versicolor* obtained from the leaves of cigar tobacco. Compounds **11** and **12** showed strong activity against MRSA ZR11, with an inhibition zone of 16.4 ± 2.2 and 18.5 ± 2.5 mm, respectively [40].

(±)-trichodermatrione A (**13**), a pair of cyclobutane-containing enantiomers with an undescribed tricyclic 6/4/6 skeleton, was isolated from *Trichoderma* sp. EFT2, an endophytic fungus from *Euonymus fortunei*. Compound **13** and enantiomers showed activity against phytopathogenic bacteria *Xanthomonas oryzae* pv. *oryzae* and *X. oryzae* pv. *oryzicola*, with the same MIC value of 64 µg/mL [41].

#### 2.1.2. Xanthones and Benzophenone Derivatives

New xanthone dimers, subplenone A (**14**), E (**15**), and G (**16**), were isolated from the fungus *Subplenodomus* sp. CPCC 401465, which resides within the Chinese medicinal plant *Gentiana straminea*. These compounds displayed remarkable inhibitory activity against MRSA, with an MIC of 0.25 μg/mL, and values ranging from 0.5 to 1.0 μg/mL against vancomycin-resistant *Enterococcus faecium* (VRE) [42].

Two novel heterodimeric tetrahydroxanthones, aflaxanthones A (**17**) and B (**18**), dimerized via an unprecedented *7*,*7′*-linkage in an sp^3^-sp^3^ dimeric manner, were isolated from the fungus *Aspergillus flavus* QQYZ obtained from a fresh blade of the mangrove *Kandelia candel*. They displayed moderate antibacterial activities against several bacteria with MIC values in the range of 12.5–25 μM [43].

Three new pestalone-type benzophenones, pestalotinones A–C (**19**−**21**), were isolated from the fungus *Pestalotiopsis trachicarpicola* SCJ551 obtained from fresh healthy stems of *Blechnum orientale*. They exhibited potent activity against *S. aureus* and MRSA (MIC: 1.25–2.5 μg/mL) [44].

One new benzophenone derivative, pseudocercone A (**22**), and two spirocyclic polyketides, pseudocercone B (**36**) and C (**77**), were isolated from the fungus *Pseudocercospora* sp. TSS-1 obtained from *Lycopodiastrum casuarinoides*. Compounds **22** and **77** displayed significant selective antibacterial activity against *S. aureus,* with MIC values of 7.8 and 3.9 μg/mL, respectively, while compound 36 showed weak activity (MIC = 62.5 μg/mL) [45].

#### 2.1.3. Pyrones and Lactones

Pannorin C (**23**) was isolated from the fungus *Aspergillus cristatus* 2H1 obtained from *Bryophyllum pinnatum*. It showed weak antibacterial activity against *S. aureus* (MIC of 20 µg/mL) [46].

The fusaritricins (**24**–**27**) exhibited antibacterial activity against plant pathogen *Pseudomonas syringae* pv. *actinidiae* (Psa) with MIC values of 128, 128, 128, and 64 μg/mL, respectively. They were obtained from the kiwi endophytic fungus *Fusarium tricinctum*. The bacterium Psa causes canker disease in kiwi fruit plantations [47].

Two undescribed isocoumarins, sporulactones E (**28**) and F (**29**), were isolated from the kiwi endophytic fungus *Paraphaeosphaeria sporulosa*. They showed antibacterial activity against *Pseudomonas syringae* pv. *actinidiae* (Psa) with an MIC value of 25 and 50 μg/mL, respectively [48].

Eutyscoparol H (**30**) and I (**31**) exhibited significant antibacterial activity against *S. aureus* and MRSA cells with an MIC value of 6.25 μg/mL; they were isolated from the fungus *Eutypella scoparia* SCBG-8 of the plant *Leptospermum brachyandrum* [49].

The (±)-isothielavic acid (**32**) demonstrated potential inhibitory effects against *Bacillus subtilis*, *Pseudomonas aeruginosa*, and *Escherichia coli*, with inhibition zones of 7 mm, 9 mm, and 10 mm, respectively; it was isolated from the fungus *Thermothielavioides terrestris* YB4 obtained from the rhizome of *Stemona mairei* [50].

Beshanzoide E (**33**) was obtained from the fungus *Penicillium commune* P-4-1 isolated from the fresh trunk bark of the critically endangered conifer *Abies beshanzuensis*. It showed antimicrobial activity against *S. aureus* (MIC,16 µg/mL) [51].

Isotalaroflavone (**34**) was obtained from the *Cercis chinensis*-derived fungus *Alternaria alternata* ZHJG5. Compound **34** was active against the phytopathogenic bacteria *Xanthomonas oryzae* pv. *oryzae*, *Xanthomonas oryzae* pv. *oryzicola*, and *Ralstonia solanacearum* with MIC values of 16, 64, and 64 µg/mL, respectively [52].

Aspergillone A (**84**), B (**85**), and D (**35**) were isolated from the fungus *Aspergillus sclerotiorum* obtained from fresh tubers of *Pinellia ternate*. Compounds **84**, **85**, and **35** showed mild antibacterial activity against *Bacillus subtilis* with MIC values of 25.79 ± 0.62, 26.41 ± 2.13, and 25.82 ± 1.35 µg/mL, respectively [53].

Two new phthalide derivatives, (−)-3-carboxypropyl-7-hydroxyphthalide (**37)** and (−)-3-carboxypropyl-7-hydroxyphthalide methyl ester (**38**), were isolated from the fungus *Penicillium vulpinum* obtained from the Chinese medicinal plant *Sophora tonkinensis*. Compound **37** exhibited medium inhibition against *Shigella dysenteriae* and *Enterobacter areogenes* with an MIC value of 12.5 µg/mL, against *Bacillus subtilis* with an MIC value of 25 µg/mL, and against *Bacillus megaterium* and *Micrococcus lysodeikticus* with an MIC value of 50 µg/mL; 38 showed medium inhibition against *E. areogenes* with an MIC value of 12.5 µg/mL [54].

Three new β-resorcylic acid lactones, including 4-O-desmethyl-aigialomycin B (**39**) and penochrochlactones C (**40**) and D (**41**), were isolated from a mycelial solid culture of the fungus *Penicillium ochrochloron* SWUKD4.1850 from the medicinal plant *Kadsura angustifolia*. Compounds **39**–**41** exhibited moderate activities against *S. aureus*, *B. subtilis*, *E. coli*, and *P. aeruginosa,* with MIC values between 9.7 and 32.0 μg/mL [55].

Penicimenolide G (**42**) is an unusual 12-membered resorcylic acid lactone ring isolated from the fungus *Aspergillus giganteus* obtained from a leaf of *Capparis carandas*. Compound **42** exhibited excellent antibacterial activity against amoxicillin-resistant strains, specifically *Bacillus cereus* (9 mm, 1 mg/disk) and *Klebsiella* sp. (8 mm, 1 mg/disk) [56].

#### 2.1.4. Depsidones and Diphenyl Ether Derivatives

Emeguisin D (**43**) was isolated from the fungus *Aspergillus unguis* BCC54176 from a leaf of coriander, *Coriandrum sativum*. It was active against *B. cereus* and *S. aureus* with an MIC value of 1.56 μg/mL [57].

Aspergillone A (**44**) exhibited moderate antibacterial activities against *B. subtilis* and *S. aureus* with MIC_50_ values of 8.5 and 32.2 μg/mL, respectively; it was isolated from the fungus *Aspergillus cristatus* from *Pinellia ternate* [58].

Mollicellins V-Y (**45**–**48**) were isolated from the fungus *Chaetomium brasiliense* obtained from stems of Thai rice. Compounds **45** and **46** exhibited strong antibacterial activity against Gram-positive bacteria *B. cereus* and *B. subtilis* with MIC values of 4–8 μg/mL, which are close to that of the standard drug kanamycin (MIC value of 2 μg/mL). Compounds **47** and **48** showed moderate antibacterial activity against different strains of MRSA with MIC values of 32–128 μg/mL, which are close to those of a standard drug, oxacillin (MIC values of 32–128 μg/mL) [59].

Rhexocerin A (**49**), rhexocerin E (**50**), rhexocercosporin A-E (**51**–**55**) and G (**56**), and rhexocerdepside A (**88**) were isolated from the fungus *Rhexocercosporidium* sp. Dzf14 obtained from the medicinal plant *Dioscorea zingiberensis* [60,61]. Compounds **49** and **50** featured an unprecedented tetracyclic carbon skeleton (6/7/5/6). Compounds **49** and **51**−**55** were active against MRSA (MIC value of 4–32 μg/mL) and vancomycin-resistant *E. faecalis* (MIC value of 4–16 μg/mL) [61]. Compounds **50**, **56**, and **88** showed weak activity against *B. subtilis* with an MIC value of 64 μg/mL [60].

Talaromurolide A (**57**) exhibited inhibitory activities against *S. aureus*, *E. coli*, and *Salmonella typhimurium* with an MIC value of 64 μg/mL; it was isolated from the fungus *Talaromyces muroii* YIMF00209 obtained from the roots of *Gmelina arborea* [62].

Epicoccethers K−N (**58**−**61**) were isolated from the fungus *Epicoccum sorghinum* derived from *Myoporum bontioides*. They showed moderate or weak antibacterial activity (MIC value of 25–200 μg/mL) toward *S. aureus* and *E. coli* O78 [63].

#### 2.1.5. Naphthalene Derivatives

Dalesconosides A–D (**62**–**65**) and F (**66**) were isolated from the fungus *Daldinia eschscholzii* MCZ-18 derived from the Chinese mangrove *Ceriops tagal*. Compounds **62**–**66** showed antibacterial activity against at least one of the bacterial strains tested (*P. aeruginosa*, *E. faecalis*, MRSA, or *E. coli*) with an MIC value of 6.25 to 50 μg/mL; in particular, **62** was the most potent [64].

Phyligustricin C (**67**) and D (**68**) were isolated from the fungus *Phyllosticta ligustricola* HDF-L-2 obtained from the conifer *Pseudotsuga gaussenii*. They exhibited antibacterial activity against *S. aureus*, each with an MIC value of 16 μg/mL [65].

3-methoxy-5-methylnaphthalene-1,7-diol (**69**) was isolated from the fungus *Diaporthe* sp. obtained from the plant *Syzygium cordatum*. Compound **69** had palpable antibacterial activities against two bacterial pathogens of beans, *Pseudomonas syringae* pv*. phaseolicola and Xanthomonas axonopodis* pv. *phaseoli,* with MIC values of 2.50 mg/mL (7.00 ± 0.00 mm) and 1.25 mg/mL (7.67 ± 0.33 mm), respectively [66].

1-(3-hydroxy-1-(hydroxymethyl)-2-methoxy-6-methylnaphthalen-7-yl)propan-2-one (**70**) and 1-(3-hydroxy-1-(hydroxymethyl)-6-methylnaphthalen-7-yl)propan-2-one (**71**) were isolated from the fungus *Phomopsis fukushii* derived from the roots of *Nicotiana tabacum*. They showed weak inhibition against MRSA with inhibition zone diameters of 10.2 ± 1.8 and 11.3 ± 2.0 mm, respectively [67].

#### 2.1.6. Other Polyketides

The 4-(5,7-dimethoxy-4-oxo-4H-chromen-2-yl)butanoic acid methyl ester (**72**) was isolated from the fungus *Penicillium sclerotiorum* MPT-250 obtained from the stems of *Taxus wallichiana* var. *chinensis* and exhibited significant antibacterial activity against carbapenem-resistant *P. aeruginosa* (MIC, 3.13 μg/mL) [68].

Stagonosporopsin C (**73**) exhibited moderate inhibitory activity against *S. aureus* with an MIC_50_ of 41.3 μM; it was isolated from the fungus *Stagonosporopsis oculihominis* obtained from *Dendrobium huoshanense*. [69].

2,6-dimethyl-5-methoxyl-7-hydroxylchromone (**74**) was isolated from the fungus *Xylomelasma* sp. Samif07 derived from the medicinal plant *Salvia miltiorrhiza*. It showed activity against the plant pathogenic bacterium *Erwinia carotovora* with an MIC value of 100 μg/mL [70].

Lawsozaheer (**75**) showed highly selective activity against the bacterium *S. aureus* (NCTC 6571) with 84.26% inhibition at 150 μg/mL, comparable to that of the standard drug ofloxacin (87.013% inhibition at 100 μg/mL). It was isolated from the fungus *Paecilomyces variotii* obtained from *Lawsonia alba* [71].

Koninginin W (**76**) was isolated from the fungus *Trichoderma koningiopsis* YIM PH30002 of *Panax notoginseng* and presented weak antibacterial activity against *E. coli*, *B. subtilis,* and *Salmonella typhimurium* with MIC values of 128, 128, and 64 μg/mL, respectively [72].

1-(8-methoxy-3-methyl-1*H*-isochromen-6-yl)propan-1-one (**78**) and 3-hydroxy-1-(8-methoxy-3-methyl-1*H*-isochromen-7-yl)propan-1-one (**79**) were isolated from a cigar tobacco-derived endophytic fungus *Aspergillus fumigatus*. Compounds **78** and **79** showed activity against *Pseudomonas syringae pv. angulata* (the main pathogenic source of tobacco angular leaf spot disease) with MIC_50_ values of 6.8 and 8.4 μg/mL, respectively [73].

Pestallic acid T (**80**) was isolated from the fungus *Pestalotiopsis trachicarpicola* SC-J551 derived from the fern *Blechnum orientale* and showed moderate activity against *S. aureus* and MRSA with an MIC value of 20 μg/mL [74].

Atrovinol (**81**) exhibited moderate inhibitory activity against *S. aureus* and *B. subtilis* with MIC values of 8.0 μg/mL and 16.0 μg/mL, respectively. It was isolated from the fungus *Trichoderma atroviride* HH-01 derived from *Illigera rhodantha* [75].

Two new tetraketide-derived phenol rhamnosides, botryrhamnosides A (**82**) and B (**83**), and a new rhamnosylated tryptophol alkaloid (botryrhamnoside C, **106**) were isolated from the fungus *Botryosphaeria dothidea* LE-07 obtained from the leaves of the rare medicinal plant Chinese tulip tree (*Liriodendron chinense*). Compounds **82**, **83**, and **106** displayed antibacterial activity against *E. coli* with MIC values of 8.0, 8.0, and 16 μg/mL, respectively [76].

Penioctadecatrienoic A (**86**) was isolated from the fungus *Penicillium pinophilum* J70 derived from the fresh leaves of *Hypericum japonicum* Thumb and exhibited a weak antibacterial effect against *S. aureus* with an MIC value of 32 μg/mL [77].

Pinophol A (**87**) was isolated from the fungus *Talaromyces pinophilus* obtained from the aerial parts of *Salvia miltiorrhiza*. It exhibited weak antibacterial activity against *Bacterium paratyphosum* B with an MIC value of 50 μg/mL [78].

#### 2.1.7. Nitrogen-Containing Compounds

The new polycyclic tetramic acids displaying a *cis*-decalin ring, epicolidines B (**89**) and C (**90**), were isolated from the mullein plant endophyte *Epicoccum* sp. 1-042. Both compounds showed promising activity against sensitive *Enterococcus faecium* strain (64 and 8 µg/mL) and vancomycin-resistant *Enterococcus faecium* (16 and 2 µg/mL), *S. aureus* (64 and 2 µg/mL), and MRSA (16 and 2 µg/mL) [79].

The citrinin derivatives, perinadine D (**91**) and perinadine E (**92**), were isolated from *Penicillium citrinum* GZWMJZ-836 associated with *Drynaria roosii*. They displayed antibacterial activities against *Bacillus subtilis* (125 and 125 µM) and different MRSA strains (62.5 to 125 µM) [80].

The unprecedented sulfur-containing heterodimers of cytochalasan and curvularin, sucurchalasins A (**93**) and B (**94**), were isolated from *Aspergillus spelaeus* GDGJ-286 obtained from the roots of *Sophora tonkinensis*; these compounds exhibited antibacterial effects against *E. faecalis* (3.1 and 3.1 µg/mL) and *B. subtilis* (6.3 and 6.3 µg/mL). These compounds represent the first examples of cytochalasin heterodimers characterized by a thioether bridge between aspochalasin and curvularin macrolide units [81].

The ethyl acetate extract of the *Sophora tonkinensis* endophyte *Diaporthe* sp. GDG-118 led to the discovery of cytochalasins, where the new 21-acetoxycytochalasin J_3_ (**95**) showed inhibitory activity against *B. anthraci* (12.5 µg/mL) and *E. coli* (12.5 µg/mL). This was the first report of *Diaporthe* sp. isolated from *S. tonkinensis*, a plant traditionally used in Chinese medicine [82].

Xylarchalasin A (**96**) and B (**97**) showed antibacterial activities against *B. subtilis* (100 and 25 µg/mL) and *E. coli* (50 and 12.5 µg/mL); they were isolated from *Xylaria* sp. GDGJ-77B derived from *Sophora tonkinensis* [83].

Among the compounds isolated from the mangrove-endophyte *Xylaria arbuscula* QYF, two undescribed cytochalasins were isolated, including the rare hydroxyperoxide, xylariachalasin B (**98**) and xylariachalasin C (**99**). Compound **98** showed antibacterial activity against MRSA (25 µM), *S. aureus* (12.5 µM), and *P. aeruginosa* (25 µM), while compound 99 showed antibacterial activity against MRSA (50 µM) and *S. typhimurium* (25 µM) [84].

A set of new cytochalasins and analogs was isolated from *Phomopsis* sp. xz-18 associated with the stems of *Camptotheca acuminata*, where phomopchalasin C_3_ (**100**) and phomopchalasin C_4_ (**101**) had conjugated diene structures in the macrocycle. Compound **100** showed antibacterial activity in a disk diffusion assay against *B. pumilus* (9 mm), and compound 101 against *B. pumilus* (8 mm) and *B. subtilis* (7 mm) [85].

A novel 1,4-oxazine-xanthone derivative, fusarioxazin (**102**), was isolated from the ethyl acetate extract of *Fusarium oxysporum* derived from *Vicia faba* and exhibited antibacterial activity against *S. aureus* (14.8 ± 0.19 mm), *B. cereus* (18.9 ± 0.72 mm), and *E. coli* (9.1 ± 1.61 mm). Oxazimes are heterocyclic compounds with oxygen and nitrogen atoms and have attracted special attention because they exhibit beneficial bioactivities and represent a class of synthetic and natural products [86].

The new isoquinolines, 1-(8-methoxy-3-methylisoquinolin-6-yl)propan-1-one (**103**) and 3-hydroxy-1-(8-methoxy-3-methylisoquinolin-6-yl)propan-1-one (**104**), were isolated from *Aspergillus puniceus* associated with cigar tobacco. Compounds **103** and **104** exhibited antibacterial activity against *P. syringae* pv. *angulata* (MIC_50_ 8.5 and 5.4 µg/mL), which is the main pathogenic source of angular leaf spot disease [87].

Peniazaphilone A (**105**) was obtained among other azaphilone derivatives by co-culturing two mangrove-endophytic *Penicillium sclerotiorum* strains, both associated with the fresh flowers of the mangrove *Aegiceras corniculatum*. This compound displayed antibacterial activity against *B. subtilis* (12.5 µM), *P. aeruginosa* (12.5 µM), and MRSA (12.5 µM) [88].

The indole alkaloids 6-(4-methoxyphenoxy)-4-methoxy-2-methyl-1*H*-indole (**107**) and 6-(3,5-dimethoxyphenoxy)-4-methoxy-2-methyl-1*H*-indole (**108**) were isolated from *Aspergillus oryzae* associated with cigar tobacco. Compounds 107 and 108 displayed potential antibacterial activity against MRSA (22.4 ± 2.4 and 24.6 ± 2.2 mm) [89].

A series of imidazole alkaloids, named fusaritricine B (**109**), C (**110**), and I (**111**), were isolated from the kiwi-associated *Fusarium tricinctum*. They showed potent activity against *Pseudomonas syringae* pv. *actinidae* with an MIC value of 50 µg/mL [90].

The new N-methoxy-1-pyridone alkaloid, chromenopyridin A (**112**), was isolated from *Penicillium nothofagi* P-6 derived from the bark of the conifer *Abies beshanzuensis*. Compound **112** exhibited activity against *S. aureus* with an MIC value of 62.5 µg/mL [91].

Alternarin A (**113**) is a new cyclic peptide isolated from the ethyl acetate extract of *Alternaria* sp. RW-AL obtained from the fruits of *Areca catechu*. Compound **113** presented activity against *B. subtilis* (17.52 ± 0.41 mm) [92].

#### 2.1.8. Terpenoids

Terpenes and terpenoids represent the most diverse class of natural products, displaying a wide range of biological activities, including antimicrobial effects, though their modes of action are not yet fully understood. There are examples of terpenes with antimicrobial activity, such as carvacrol, thymol, menthol and geraniol, linalyl acetate, (+)-carvone, trans cinnamaldehyde, and bonianic acid A and B, while others like farnesol or xylitol can be used in combination with antibiotics (amoxicillin, doxycycline, oxacillin, vancomycin) to improve antimicrobial action [93].

The new andrastatin derivative, 10-demethylated andrastone A (**114**), was isolated from the endophytic fungus *Penicillium vulpinum* derived from the roots of *Sophora tonkinensis*. It showed antibacterial activity against *B. megaterium* with an MIC value of 6.25 µg/mL. The andrastatin scaffold is a 6,6,6,5-tetracarbocyclic skeleton and is rarely found in nature [94].

The new helvolic acid derivatives, sarocladilactone A (**115**) and B (**116**), were isolated from the *Oryza rufipogon* endophytic fungus *Sarocladium oryzae* DX-THL3. Compound **115** exhibited antibacterial activities against *S. aureus* (64 µg/mL), and compound 116 against *S. aureus* (4 µg/mL) and *E. coli* (64 µg/mL). Helvolic acid is a fusidane-type antibiotic, which displays potent activity against Gram-positive bacteria and has attracted renewed attention for its lack of cross-resistance with other antibiotics [95].

Four new ergosterol derivatives, ganodermasides E-H (**117**–**120**), were isolated from the endophytic *Epicoccum poae* DJ-F associated with *Euphorbia royleana*. Their antibacterial activity was tested against *S. aureus* (1.7, 2.1, 0.9, and 1.6 mM), and phytopathogens *Pseudomonas syringae* pv. *tabaci* (3.3, 1.0, 0.9, and 1.6 mM) and *Ralstonia solanacearum* (3.3, 1.0, 0.4–0.9, and 0.8–1.6 mM). The *Epicoccum* genus is well known for its capability to produce bioactive compounds with potential as biocontrol agents against many phytopathogens [96].

The study of endophyte *Arthrinium* sp. ZS03 associated with *Acorus tatarinowii* yielded a set of pimarane diterpenoids, including seven new arthrinoids, of which arthrinoid G (**121**) exhibited activity against *Klebsiella pneumonia* with an MIC value of 8 µg/mL*,* comparable to the reference antibiotic amikacin (2 µg/mL). Compound **121** also showed activity against *E. coli* (16 µg/mL), *P. aeruginosa* (64 µg/mL), and *Acinetobacter baumannii* (64 µg/mL) [97].

Among the undescribed eremophilane derivatives isolated from *Diaporthe* sp. BCC69512 obtained from the leaf of *Etlingera littoralis*, diaportheremophilin B (**122**) and E (**123**) showed broad antimicrobial activity against *M. tuberculosis* (50 and 25 µg/mL) and *B. cereus* (25 and 25 µg/mL). This was the first report of eremophilane sesquiterpenoids bearing ester linkage at C-1 in the genus *Diaporthe* [98].

The chemical investigation of kiwi (*Actinidia chinensis*) endophytic fungus *Bipolaris* sp. led to the isolation of novel sativane sesquiterpenoids containing additional skeletal carbons, including the dimers bipolarisorokin M (**124**) and N (**125**). Both compounds showed certain inhibitory activity against phytopathogen *P. syringae* pv. *actinidae* with MIC values of 32 and 64 μg/mL, respectively [99].

The mycelial extract of *Acremonium* sp. MNA-F-1 derived from the inner tissue of *Pimpnella anisum* roots led to the isolation of the undescribed prenylated chlorophenol, acremochlorin S (**126**), along with other related derivatives. Compound **126** revealed moderate antibacterial activity against *S. aureus* with an MIC_90_ value of 62.5 mM [100].

The overexpression of the pathway-specific transcription factor LutB from the endophytic fungus *Talaromyces* sp. XSJC-F4, isolated from *Palhinhaea cernua*, led to the production of new sclerotiorin-type azaphilones, including one named dehydrated luteusin E (**127**), which exhibited activity against *B. subtilis* with an MIC value of 64 μg/mL [101].

Eight new sesquiterpenes called eutyscoparins were isolated from *Eutypella scoparia* SCBG-8 associated with the leaves of *Leptospermum brachyandrum*. Eutyscoparin G (**128**) was the only compound active in the antibacterial assay against *S. aureus* and MRSA, with an MIC value of 6.25 μg/mL [102].

The gymnomitrane-type sesquiterpenes, xylariacinol A (**129**) and D (**130**), were isolated from the fungus *Annulohypoxylon* sp. KYG-19 obtained from the leaves of *Illigera celebica*. Compounds **129** and **130** displayed activities against *E. coli* (4 and 2 μg/mL), *B. subtilis* (32 and 16 μg/mL), and *S. aureus* (both, 32 μg/mL). Gymnomitrane-type sesquiterpene has attracted the attention of scientists due to its multiple pharmacological activities and complex structures [103].

Among a set of fourteen eremophilane sesquiterpenoids isolated from *Septoria rudbeckiae* associated with *Karelinia caspia*, the new septoeremophilane named septoeremophilane D (**131**) presented an effect against *P. syringae* pv. *actinidae* (6.25 μM), *B. cereus* (6.25 μM), and MRSA (50 μM). These septoeremophilanes feature a highly oxygenated structure with a 6/6/5 tricyclic system and a hemiacetal moiety [104].

The new caryophyllene-type sesquiterpene, punctaporonin T (**132**), was isolated from *Chaetomium globosum* TC2-041 obtained from the leaves of *Empetrum nigrum.* Compound **132** showed weak inhibitory activity against *M. tuberculosis* (IC_50_ = 36.8 µg/mL) and *S. aureus* (IC_50_ = 83.0 µg/mL). This was the first report of a caryophyllene sesquiterpenoid isolated from *C. globosum* [105].

## 3. Discussion

It is a fact that the discovery and application of antibiotics to treat infectious diseases played an important role in assuring human and animal health; however, their intensified and inappropriate use has led to the outbreak of pathogens with antimicrobial and multidrug resistance. This situation has created the need for novel antimicrobial agents; thus, endophytic fungi have proved to be a promising alternative.

The World Health Organization, in its 2024 Bacterial Priority Pathogens List to guide research, development, and strategies to prevent and control antimicrobial resistance, grouped antibiotic-resistant pathogens into three categories: (a) the critical group which includes carbapenem-resistant *Acinetobacter baumannii* and third-generation cephalosporin-resistant Enterobacterales, also carbapenem-resistant Enterobacterales; (b) the high group which comprises fluoroquinolone-resistant *Salmonella* Typhi, fluoroquinolone-resistant *Shigella* spp., vancomycin-resistant *Enterococcus faecium*, carbapenem-resistant *Pseudomonas aeruginosa*, and non-typhoidal fluoroquinolone-resistant *Salmonella*; and (c) the medium group, which involves two subcategories, namely, (c.1) group A, consisting of macrolide-resistant *Streptococci*, macrolide-resistant *Streptococcus pneumoniae*, and ampicillin-resistant *Haemophilus influenzae*, and (c.2) group B, consisting of penicillin-resistant *Streptococci*.

**Figure 11:**
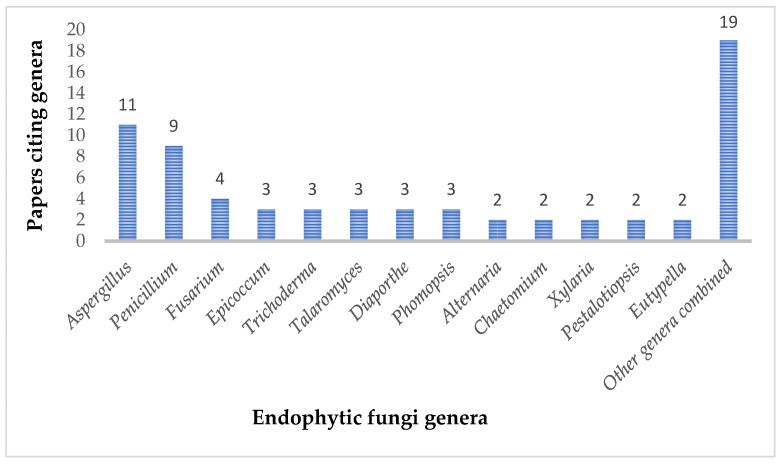
Number of papers citing antibacterial compounds isolated from endophytic fungi genera.

In this review, a total of 132 secondary metabolites from endophytic fungi showed antibacterial activity against a group of bacteria, including antibiotic-resistant strains such as methicillin-resistant *S. aureus* (MRSA), carbapenem-resistant *P. aeruginosa*, vancomycin-resistant *Enterococcus faecalis*, and vancomycin-resistant *Enterococcus faecium*. We found that the compounds were isolated from a variety of endophytic fungi genera, confirming them as a promising source for antimicrobial agents; *Aspergillus* and *Penicillium* were the most cited genera (Figure 11).

**Figure 12:**
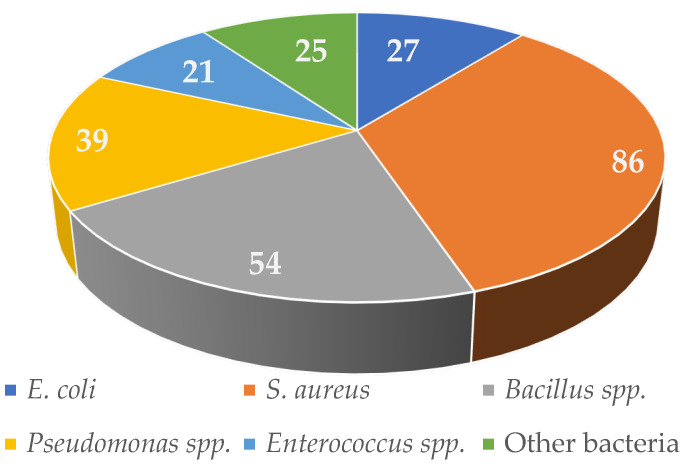
The proportion of reported antimicrobial endophytic compounds against bacteria.

The data revealed that 27 compounds exhibited notable antimicrobial effects on *E. coli*, 86 on *S. aureus*, 54 on *Bacillus* spp., 39 on *Pseudomonas* spp., 21 on *Enterococcus* spp., and 25 on other bacterial taxa (Figure 12).

The data analyzed also indicated that the antimicrobial assays employed standard antibiotics as positive controls, which may provide insights into the potential mechanisms of action of the tested metabolites. The following antibiotics were used: penicillin G, vancomycin, cefradine, oxacillin, ampicillin, amoxicillin, and fosfomycin (inhibitors of cell wall synthesis); ciprofloxacin, levofloxacin, moxifloxacin, ofloxacin, and rifampicin (inhibitors of nucleic acid synthesis); amikacin, kanamycin, gentamicin, streptomycin, tobramycin, and chloramphenicol (inhibitors of protein synthesis); and daptomycin (disruptor of plasma membrane integrity) [106,107].

Compound parengyomarin A (**1**) exhibited greater potency (MIC: 0.39 µM) than the reference drug moxifloxacin (MIC: 0.78 µM) against *Staphylococcus aureus*. A notable effect was also observed against methicillin-resistant *S. aureus* (MRSA), with an MIC of 0.39 µM, compared to 6.25 µM for the control. Moreover, the compounds dothideomin A (**3**), dothideomin C (**5**), and dothideomin D (**6**) displayed antimicrobial activity in the range of 0.4–1.6 µg/mL, closely approximating that of chloramphenicol (0.3–1.5 µg/mL), suggesting a potential structure–activity relationship. Additionally, compounds **3**, **5**, and **6** showed inhibitory activity against *Bacillus subtilis* within a similar range (0.4–1.6 µg/mL), aligning with the reference compound’s activity (0.3–1.5 µg/mL). The inhibitory activities of **3**, **5**, and **6** were stronger than those of dothideomin B (**4**) (ketone carbonyl was reduced), which indicated that the intact anthraquinone backbone played an important role in antibacterial activity.

The compounds botryrhamnoside A (**82**) and botryrhamnoside B (**83**), along with the tricyclic diterpenes xylariacinol A (**129**) and xylariacinol D (**130**), demonstrated notable activity against *Escherichia coli*, with MIC values comparable to those of the positive controls (chloramphenicol and kanamycin, respectively). Compound **104** displayed potent activity against *Pseudomonas syringae* pv. *angulata* (MIC_50_: 5.4 µg/mL), relative to the agricultural control streptomycin (2.2 µg/mL). Compounds mollicellin V (**45**) and mollicellin W (**46**) showed strong activity against *Bacillus cereus* (MIC: 4 µg/mL), compared to the reference antibiotic kanamycin (2 µg/mL). Finally, Alternarin A (**113**) exhibited an inhibition zone of 17.52 ± 0.41 mm against *Bacillus subtilis*, comparable to the 20.18 ± 0.34 mm zone observed for ampicillin.

While in vitro antibacterial assays provide rapid and cost-effective preliminary data on antimicrobial activity, they often fail to replicate the complex biological conditions of a living host. Factors such as immune response, bioavailability, and metabolic stability are not accounted for in vitro [108,109]. Consequently, promising in vitro results may not translate to therapeutic efficacy in vivo. Therefore, in vivo validation is essential to confirm clinical relevance and ensure the safe and effective development of antibacterial agents [110].

## 4. Materials and Methods

The search was initially conducted in Scifinder^®^ using the term “endophytic fungi”; then we used different filters such as document type (journal and review), language (English), publication year (2021 to 2024), and concept (antibacterial agents), obtaining 334 total hits. After removing duplicates, we selected articles related to the aim of this review, resulting in 72 articles describing new natural products with antibacterial activity.

## 5. Conclusions

The 132 secondary metabolites identified from endophytic fungi exhibit notable antibacterial activity, including efficacy against high-priority drug-resistant pathogens such as MRSA, carbapenem-resistant *Pseudomonas aeruginosa*, and vancomycin-resistant *Enterococcus* spp., highlighting their potential as alternative sources for novel antimicrobial agents. The data presented in this review suggest that a subset of these compounds may serve as candidates for further studies on synergistic effects in combination with traditional antibiotics and structure–activity relationship analysis.

## Figures and Tables

**Table 1:** Selected antimicrobial secondary metabolites isolated from endophytic fungi.

Compound	Antimicrobial Activity (μg/mL) *	Positive Control (μg/mL) *
parengyomarin A (**1**)	*S. aureus* 0.39 * MRSA 0.39 *	moxifloxacin 0.78 * moxifloxacin 6.25 *
dothideomin A (**3**)	*B. subtilis* 0.4 *S. aureus* 0.4	chloramphenicol 0.3–1.5
dothideomin C (**5**)	*B. subtilis* 0.4 *S. aureus* 0.4	chloramphenicol 0.3–1.5
dothideomin D (**6**)	*S. aureus* 1.6	chloramphenicol 0.3–1.5
subplenone A (**14**)	ATCC 700698 MRSA 0.25 vancomicin-resistant *E. faecalis* 2.0	levofloxacin 0.125 levofloxacin 1.0
subplenone E (**15**)	ATCC 700698 MRSA 0.25 vancomicin-resistant *E. faecalis* 2.0	levofloxacin 0.125 levofloxacin 1.0
pestalotinone A (**19**)	MRSA 2.5	kanamycin 1.25
mollicellin V (**45**)	ATCC 25923 MRSA 64	oxacillin 32–128
mollicellin W (**46**)	*B. subtilis* 4.0 ATCC 25923 32	kanamycin 2.0 oxacillin 32–128
rhexocerin (**50**)	*B. subtilis* 64	streptomycin sulphate 32
rhexocercosporin E (**55**)	MRSA T144 4.0	vancomycin 2.0
epicoccether K (**58**)	*E. coli* serotype 06 25	cefradine 12.5
4-(5,7-dimethoxy-4-oxo-4H-chromen-2-yl)butanoic acid methyl ester (**72**)	carbapenem-resistant *P. aeruginosa* 3.13	ciprofloxacin 0.78
koninginin W (**76**)	*E. coli* 128	ampicillin 128–256
pseudocercone C (**77**)	*S. aureus* 3.9	amikacin 4.0
botryrhamnoside A (**82**)	*E. coli* 8.0	chloramphenicol 2.0
botryrhamnoside B (**83**)	*E. coli* 8.0	chloramphenicol 2.0
xylariacinol D (**130**)	*E. coli* 2.0	kanamycin 1.0

* in micromolar.

## Data Availability

No new data were created or analyzed in this study. Data sharing is not applicable to this article.

## Supplementary Materials

The following supporting information can be downloaded at https://www.mdpi.com/article/10.3390/antibiotics14070644/s1, Table S1: Antibacterial activity of compounds against *E. coli*; Table S2: Antibacterial activity of compounds against different *Pseudomonas* strains; Table S3: Antibacterial activity of compounds against different *Bacillus* strains; Table S4: Antibacterial activity of compounds against different *Staphylococcus aureus* and MRSA strains; Table S5: Antibacterial activity of compounds against other genus strains.
