# Supplementary material for: Antibacterial Compounds Isolated from Endophytic Fungi Reported from 2021 to 2024
Source: Antibiotics (Basel). 2025 Jun 25;14(7):644. doi: 10.3390/antibiotics14070644 (PMC12291817; doi:10.3390/antibiotics14070644)
Supplement: Supplementary file 1 [file antibiotics-14-00644-s001.zip › antibiotics-3708081-supplementary.pdf]

antibiotics

Supplementary Information

Antibacterial compounds isolated from endophytic fungi reported from 2021 to 2024

Humberto E. Ortega [1,2,3], Daniel Torres-Mendoza [1,2,4] and Luis Cubilla-Rios [1,2,3,*]

[1] Departamento de Química Orgánica, Facultad de Ciencias Naturales, Exactas y Tecnología, Universidad de Panamá, Panamá 0824, Panamá; humberto.ortegad@up.ac.pa (H.E.O); daniel-t.torres-m@up.ac.pa (D.T.-M.)
[2] Laboratorio de Bioorgánica Tropical, Facultad de Ciencias Naturales, Exactas y Tecnología, Universidad de Panamá, Panamá 0824, Panamá.
[3] Sistema Nacional de Investigación (SNI), Secretaría Nacional de Ciencia, Tecnología e Innovación, Ciudad del Saber, Clayton, Panamá 0816, Panamá.
[4] Vicerrectoría de Investigación y Postgrado, Universidad de Panamá, Panamá 0824, Panamá
* Correspondence: luis.cubilla@up.ac.pa; Tel.: +507-6676-5824

 https://doi.org/10.3390/xxxxx

Table S1. Antibacterial activity of compounds against *Escherichia coli*

Compounds	Biological activity	Positive control	reference
pseudocercone A (**22**)	62.5 μg/mL	amikacin 4.0 μg/mL	[45]
pseudocercone B (**36**)	62.5 μg/mL	amikacin 4.0 μg/mL	[45]
pseudocercone C (**77**)	62.5 μg/mL	amikacin 4.0 μg/mL	[45]
(±)-isothielavic acid (**32**)	10 mm	gentamicin 19.6 mm	[50]
4-O-desmethyl-aigialomycin B (**39**)	19.6 ± 1.0 μg/mL	ciprofloxacin 0.25 ± 0.01 μg/mL	[55]
penochrochlactone C (**40**)	19.3 ± 0.4 μg/mL	ciprofloxacin 0.25 ± 0.01 μg/mL	[55]
penochrochlactone D (**41**)	17.3 ± 0.6 μg/mL	ciprofloxacin 0.25 ± 0.01 μg/mL	[55]
talaromurolide A (**57**)	64 μg/mL	chloramphenicol 16 μg/mL	[62]
epicoccether K (**58**)	serotype 06 25 μg/mL<br>serotype 078 100 μg/mL	cefradine 12.5 μg/mL	[63]
epicoccether L (**59**)	serotype 06 50 μg/mL<br>serotype 078 200 μg/mL	cefradine 12.5 μg/mL	[63]
epicoccether M (**60**)	serotype 06 >200 μg/mL<br>serotype 078 100 μg/mL	cefradine 12.5 μg/mL	[63]
epicoccether N (**61**)	serotype 06 200 μg/mL<br>serotype 078 25 μg/mL	cefradine 12.5 μg/mL	[63]
dalesconoside A (**62**)	6.25 μg/mL	ciprofloxacin 0.625 μg/mL	[64]
dalesconoside C (**64**)	12.5 μg/mL	ciprofloxacin 0.625 μg/mL	[64]
dalesconoside F (**66**)	50 μg/mL	ciprofloxacin 0.625 μg/mL	[64]
koninginin W (**76**)	128 μg/mL	ampicillin 128 – 256 μg/mL<br>kanamycin 16 – 32 μg/mL	[72]
botryrhamnoside A (**82**)	8 μg/mL	chloramphenicol 2.0 μg/mL	[76]
botryrhamnoside B (**83**)	8 μg/mL	chloramphenicol 2.0 μg/mL	[76]
botryrhamnoside C (**106**)	16 μg/mL	chloramphenicol 2.0 μg/mL	[76]
21-acetoxycytochalasin J_3 (**95**)	12.5 μg/mL	ampicillin 3.125 μg/mL	[82]
xylarchalasin A (**96**)	50 μg/mL	ampicillin 1.56 μg/mL	[83]
xylarchalasin B (**97**)	12.5 μg/mL	ampicillin 1.56 μg/mL	[83]
Fusarioxazin (**102**)	9.1 ± 1.61 mm	ciprofloxacin 27.6 ± 0.78 mm	[86]
sarocladilactone B (**116**)	64 μg/mL	streptomycin sulphate 8 μg/mL	[95]
arthrinoid G (**121**)	16 μg/mL	amikacin 4 μg/mL	[97]
xylariacinol A (**129**)	4 μg/mL	kanamycin 1 μg/mL	[103]
xylariacinol D (**130**)	2 μg/mL	kanamycin 1 μg/ mL	[103]

Table S2. Antibacterial activity of compounds against different *Pseudomonas* strains

compound	Biological activity	Positive control	reference
P. aeruginosa			
6-hydroxy-astropaquinone B (**7**)	6.3 μg/mL	N.A.	[38]
astropaquinone D (**8**)	6.3 μg/mL	N.A.	[38]
pseudocercone A (**22**)	125 μg/mL	amikacin 4 μg/mL	[45]
pseudocercone B (**36**)	125 μg/mL	amikacin 4 μg/mL	[45]
(±)-isothielavic acid (**32**)	9 mm	gentamycin 19.6 mm	[50]
4-O-desmethyl-aigialomycin B (**39**)	17.9 ± 1.0 μg/mL	ciprofloxacin 0.22 ± 0.02 μg/mL	[55]
pernochrochlactone C (**40**)	32.0 ± 1.0 μg/mL	ciprofloxacin 0.22 ± 0.02 μg/mL	[55]
pernochrochlactone D (**41**)	28.1 ± 1.4 μg/mL	ciprofloxacin 0.22 ± 0.02 μg/mL	[55]
mollicellin V (**45**)	128 μg/mL	gentamicin 0.5 μg/mL	[59]
mollicellin W (**46**)	128 μg/mL	gentamicin 0.5 μg/mL	[59]
dalesconoside A (**62**)	6.25 μg/mL	ciprofloxacin 0.78 μg/mL	[64]
dalesconoside B (**63**)	50 μg/mL	ciprofloxacin 0.78 μg/mL	[64]
dalesconoside C (**64**)	25 μg/mL	ciprofloxacin 0.78 μg/mL	[64]
dalesconocide D (**65**)	25 μg/mL	ciprofloxacin 0.78 μg/mL	[64]
4-(5,7-dimethoxy-4-oxo-4H-chromen-2-yl)butanoic acid methyl ester (**72**)	carbapenem-resistant 3.13 μg/mL	ciprofloxacin 0.78 μg/mL	[68]
xylariachalasin B (**98**)	25 μM	ampicillin 0.13 μM	[84]
peniazaphilone A (**105**)	12.5 μM	ampicillin 1.6 μM	[88]
arthrinoid G (**121**)	64 μg/mL	amikacin 2 μg/mL	[97]
***Pseudomonas syringae* pv. actinidiae**			
(+)-fusaritricin A (**24**)	128 μg/mL	72% streptomycin 12.5 μg/mL	[47]
(-)-fusaritricin A (**25**)	128 μg/mL	72% streptomycin 12.5 μg/mL	[47]
fusaritricin B (**26**)	128 μg/mL	72% streptomycin 12.5 μg/mL	[47]
fusaritricin C (**27**)	64 μg/mL	72% streptomycin 12.5 μg/mL	[47]
sporulactones E (**28**)	25 μg/mL	streptomycin sulphate 12.5 μg/mL	[48]
sporulactones F (**29**)	50 μg/mL	streptomycin sulphate 12.5 μg/mL	[48]
fusaritricine B (**109**)	50 μg/mL	72% streptomycin 12.5 μg/mL	[90]
fusaritricine C (**110**)	50 μg/mL	72% streptomycin 12.5 μg/mL	[90]
fusaritricine I (**111**)	50 μg/mL	72% streptomycin 12.5 μg/mL	[90]
bipolarisorokin M (**124**)	32 μg/mL	streptomycin 8 μg/mL	[99]
bipolarisorokin N (**125**)	64 μg/mL	streptomycin 8 μg/mL	[99]
septoeremophilane D (**131**)	6.25 μM	ampicillin sodium 3.12 μM<br>fosfomycin sodium 50 μM	[104]

Table S2 (cont.).

***Pseudomonas syringae* pv. angulata**			
1-(8-methoxy-3-methyl-1H-isochromen-6-yl)propan-1-one (**78**)	MIC_{50} 6.8 µg/mL	agricultural streptomycin 2.2 µg/mL	[73]
3-hydroxy-1-(8-methoxy-3-methyl-1H-iso-chromen-7-yl)propan-1-one (**79**)	MIC_{50} 8.4 µg/mL	agricultural streptomycin 2.2 µg/mL	[73]
1-(8-Methoxy-3-methylisoquinolin-6-yl)pro-pan-1-one (**103**)	MIC_{50} 8.5 µg/mL	agricultural streptomycin 2.2 µg/mL	[87]
3-Hydroxy-1-(8-methoxy-3-methylisoquino-lin-6-yl)-propan-1-one (**104**)	MIC_{50} 5.4 µg/mL	agricultural streptomycin 2.2 µg/mL	[87]
***Pseudomonas syringae* pv phaseolicola**			
3-methoxy-5-methylnaphthalene-1,7-diol (**69**)	2.50 mg/mL (7.00 ± 0.00 mm)	N.A.	[66]
***Pseudomonas syringae* pv. tabaci**			
ganodermasides E (**117**)	3.3 mM	N.A.	[96]
ganodermasides F (**118**)	1.0 mM	N.A.	[96]
ganodermasides G (**119**)	0.9 mM	N.A.	[96]
ganodermasides H (**120**)	1.6 mM	N.A.	[96]

Table S3. Antibacterial activity of compounds against different *Bacillus* strains

compound	Biological activity	Positive control	reference
B. subtilis			
dothideomin A (**3**)	0.4 μg/mL	chloramphenicol 0.3 – 1.5 μg/mL	[37]
dothideomin B (**4**)	6.5 μg/mL	chloramphenicol 0.3 – 1.5 μg/mL	[37]
dothiodeomin C (**5**)	0.4 μg/mL	chloramphenicol 0.3 – 1.5 μg/mL	[37]
dothideomin D (**6**)	1.6 μg/mL	chloramphenicol 0.3 – 1.5 μg/mL	[37]
aflaxanthone A (**17**)	12.5 μM	ampicillin 0.39 mM	[43]
aflaxanthone B (**18**)	25 μM	ampicillin 0.39 mM	[43]
(±)-isothielavic acid (**32**)	7 mm	gentamicin 19.6 mm	[50]
aspergillone A (**84**)	25.79 ± 0.62 μg/mL	penicillin 0.34 ± 0.06 μg/mL	[53]
aspergillone B (**85**)	26.41 ± 2.13 μg/mL	penicillin 0.34 ± 0.06 μg/mL	[53]
aspergillone D (**35**)	25.82 ± 1.35 μg/mL	penicillin 0.34 ± 0.06 μg/mL	[53]
(-)-3-carboxypropyl-7-hydroxyphthalide (**37**)	25 μg/mL	ampicillin 0.78 – 3.125 μg/mL	[54]
4-O-desmethyl-aigialomycin B (**39**)	18.2 ± 1.5 μg/mL	ciprofloxacin 0.55 ± 0.07 μg/mL	[55]
penochrochlactone C (**40**)	27.2 ± 1.0 μg/mL	ciprofloxacin 0.55 ± 0.07 μg/mL	[55]
penochrochlactone D (**41**)	23.2 ± 1.4 μg/mL	ciprofloxacin 0.55 ± 0.07 μg/mL	[55]
penicimenolide G (**42**)	7 mm (1 mg/disc)<br>9 mm (1.5 mg/disc)<br>11 mm (2 mg/disc)	amoxicillin 8 mm (1 mg/disc)	[56]
aspergillone A (**44**)	MIC_{50} 8.5 μg/mL	N.A.	[58]
mollicellin V (**45**)	8 μg/mL	kanamycin 2 μg/mL	[59]
mollicellin W (**46**)	4 μg/mL	kanamycin 2 μg/mL	[59]
rhexocerin E (**50**)	64 μg/mL	streptomycin sulphate 32 μg/mL	[60]
rhexocercosporin G (**56**)	64 μg/mL	streptomycin sulphate 32 μg/mL<br>daptomycin 16 μg/mL	[60]
rhexocerdepside A (**88**)	64 μg/mL	streptomycin sulphate 32 μg/mL<br>daptomycin 16 μg/mL	[60]
koninginin W (**76**)	128 μg/mL	kanamycin 64-128 μg/mL<br>ampicillin 16-32 μg/mL	[72]
Atrovinol (**81**)	16 μg/mL	N.A.	[75]
perinadine D (**91**)	125 μM	ciprofloxacin 7.5 μM	[80]
perinadine E (**92**)	125 μM	ciprofloxacin 7.5 μM	[80]
sucurchalasin A (**93**)	6.3 μg/mL	ciprofloxacin 1.6 μg/mL	[81]
sucurchalasin B (**94**)	6.3 μg/mL	ciprofloxacin 1.6 μg/mL	[81]
21-acetoxycytochalasin J_3 (**95**)	25 μg/mL	ampicillin 3.125 μg/mL	[82]
xylarchalasin A (**96**)	100 μg/mL	ampicillin 1.56 μg/mL	[83]
xylarchalasin B (**97**)	25 μg/mL	ampicillin 1.56 μg/mL	[83]
phomopchalasin C_4 (**101**)	7 mm	gentamicin 16 mm	[85]
peniazaphilone A (**105**)	12.5 μM	ampicillin 3.2 μM	[88]
alternarin A (**113**)	17.52 ± 0.41 mm	ampicillin 20.18 ± 0.34 mm	[92]

Table S3 (cont.).

B. subtilis			
dehydrated luteusin E (**127**)	64 μg/mL	vancomycin ≤ 0.5 μg/mL	[101]
xylariacinol A (**129**)	32 μg/mL	kanamycin 1 μg/mL	[103]
xylariacinol D (**130**)	16 μg/mL	kanamycin 1 μg/mL	[103]
B. cereus			
penicimenolide G (**42**)	9 mm (1 mg/disc)<br>9.5 mm (1.5 mg/disc)<br>11 mm (2 mg/disc)	amoxicillin 0.0 mm (1 mg/disc)	[56]
emeguisin D (**43**)	1.56 μg/mL	rifampicin 0.31 μg/mL	[57]
mollicellin V (**45**)	4 μg/mL	kanamycin 2 μg/mL	[59]
mollicellin W (**46**)	4 μg/mL	kanamycin 2 μg/mL	[59]
Fusarioxazin (**102**)	18.9 ± 0.72 mm	ciprofloxacin 20.5 ± 0.29 mm	[86]
diaportheremopholin B (**122**)	25 μg/mL	vancomycin 4 μg/mL<br>rifampicin 0.625 μg/mL	[98]
diaportheremopholin E (**123**)	25 μg/mL	vancomycin 4 μg/mL<br>rifampicin 0.625 μg/mL	[98]
septoreremophilane D (**131**)	50 μM	ampicillin sodium 3.13 μM<br>fosfomycin sodium 12.5 μM	[104]
B. megaterium			
(-)-3-carboxypropyl-7-hydroxyphthalide (**37**)	50 μg/mL	ampicillin 0.78 – 3.125 μg/mL	[54]
21-acetoxycytochalasin J_3 (**95**)	50 μg/mL	ampicillin 3.125 mg/mL	[82]
xylarchalasin A (**96**)	25 μg/mL	ampicillin 1.56 μg/mL	[83]
xylarchalasin B (**97**)	50 μg/mL	ampicillin 1.56 μg/mL	[83]
10-demethylated andrastone A (**114**)	6.25 μg/mL	ampicillin 0.78 μg/mL	[94]
B. anthracis			
21-acetoxycytochalasin J_3 (**95**)	12.5 μg/mL	ampicillin 3.125 μg/mL	[82]
xylarchalasin A (**96**)	50 μg/mL	ampicillin 3.125 μg/mL	[83]
xylarchalasin B (**97**)	50 μg/mL	ampicillin 3.125 μg/mL	[83]
B. pumilus			
phomopchalasin C_3 (**100**)	CMCC 63202 9 mm	gentamicin 16 mm	[85]
phomopchalasin C_4 (**101**)	CMCC 63202 8 mm	gentamicin 16 mm	[85]

Table S4. Antibacterial activity of compounds against different *Staphylococcus aureus* and MRSA strains

compound	Biological activity	Positive control	reference
parengyomarin A (**1**)	*S. aureus* 0.39 µM<br>MRSA 0.39 µM	moxifloxacin 0.78 µM<br>moxifloxacin 6.25 µM	[36]
parengyomarin B (**2**)	*S. aureus* 1.56 µM<br>MRSA 1.56 µM	moxifloxacin 0.78 µM<br>moxifloxacin 6.25 µM	[36]
dothideomin A (**3**)	*S. aureus* 0.4 µg/mL	chloramphenicol 0.3 – 1.5 µg/mL	[37]
dothideomin B (**4**)	*S. aureus* 6.5 µg/mL	chloramphenicol 0.3 – 1.5 µg/mL	[37]
dothideomin C (**5**)	*S. aureus* 0.4 µg/mL	chloramphenicol 0.3 – 1.5 µg/mL	[37]
dothideomin D (**6**)	*S. aureus* 1.6 µg/mL	chloramphenicol 0.3 – 1.5 µg/mL	[37]
6-hydroxy-astropaquinone B (**7**)	*S. aureus* 6.3 µg/mL	N.A.	[38]
astropaquinone D (**8**)	*S. aureus* 12.5 µg/mL	N.A.	[38]
3-hydroxy-6-hydroxymethyl-2,5-dimethyl-anthraquinone (**9**)	MRSA 14.2 ± 2.0 mm	30 µg of vancomycin 32 mm	[39]
6-hydroxymethyl-3-methoxy-2,5-dime-thylanthraquinone (**10**)	MRSA 14.8 ± 2.2 mm	30 µg of vancomycin 32 mm	[39]
2´,6-dimethyl-7-methoxy-[2,3-b]furan-an-thraquinone (**11**)	MRSA 16.4 ± 2.2 mm	30 µg of vancomycin 32 mm	[40]
1,7-dimethoxy-2´,6-dimethyl-[2,3-b]furan-anthraquinone (**12**)	MRSA 18.5 ± 2.5 mm	30 µg of vancomycin 32 mm	[40]
subplenone A (**14**)	ATCC 43300 MRSA 0.5 µg/mL<br>ATCC 700698 MRSA 0.25 µg/mL<br>ATCC29213 MSSA 0.5 µg/mL	levofloxacin 0.125 µg/mL	[42]
subplenone E (**15**)	ATCC 43300 MRSA 0.5 µg/mL<br>ATCC 700698 MRSA 0.25 µg/mL<br>ATCC 29213 MSSA 0.5 µg/mL	levofloxacin 0.125 µg/mL	[42]
subplenone G (**16**)	ATCC 43300 MRSA 0.5 µg/mL<br>ATCC 700698 MRSA 0.25 µg/mL<br>ATCC29213 MSSA 0.5 µg/mL	levofloxacin 0.125 µg/mL	[42]
aflaxanthone A (**17**)	MRSA 12.5 µM	ampicillin 0.39 µM	[43]
pestalotinone A (**19**)	*S. aureus* 2.5 µg/mL<br>MRSA 2.5 µg/mL	Kanamycin 1.25 µg/mL<br>Vancomycin 0.625 µg/mL	[44]
pestalotinone B (**20**)	*S. aureus* 1.25 µg/mL<br>MRSA 1.25 µg/mL	Kanamycin 1.25 µg/mL<br>Vancomycin 0.625 µg/mL	[44]
pestalotinone C (**21**)	*S. aureus* 2.5 µg/mL<br>MRSA 2.5 µg/mL	Kanamycin 1.25 µg/mL<br>Vancomycin 0.625 µg/mL	[44]
pseudocercone A (**22**)	*S. aureus* 7.8 µg/mL	amikacin 4 µg/mL	[45]
pseudocercone B (**36**)	*S. aureus* 62.5 µg/ml	amikacin 4 µg/mL	[45]
pseudocercone C (**77**)	*S. aureus* 3.9 µg/mL	amikacin 4 µg/mL	[45]
pannorin C (**23**)	*S. aureus* 20 µg/mL	N.A.	[46]
eutyscoparol H (**30**)	*S. aureus* and MRSA 6.25 µg/mL	vancomycin 1.25 µg/mL	[49]
eutyscoparol I (**31**)	*S. aureus* and MRSA 6.25 µg/mL	vancomycin 1.25 µg/mL	[49]

Table S4 (Cont.).

beshanzoide E (**33**)	*S. aureus* 16 μg/mL	N.A.	[51]
(-)-3-carboxypropyl-7-hydroxyphthalide (**37**)	*S. aureus* 25 μg/mL	ampicillin 0.78 – 3.125 μg/mL	[54]
4-O-desmethyl-aigialomycin B (**39**)	*S. aureus* 12.3 ± 0.7 μg/mL	ciprofloxacin 0.23 ± 0.02 μg/mL	[55]
penochrochlactone C (**40**)	*S. aureus* 13.6 ± 1.4 μg/mL	ciprofloxacin 0.23 ± 0.02 μg/mL	[55]
penochrochlactone D (**41**)	*S. aureus* 9.7 ± 0.6 μg/mL	ciprofloxacin 0.23 ± 0.02 μg/mL	[55]
emeguisin D (**43**)	*S. aureus* 1.56 μg/mL	rifampicin 0.08 μg/mL	[57]
aspergillone A (**44**)	*S. aureus* MIC_{50} 32.2 μg/mL	N.A.	[58]
mollicellin V (**45**)	ATCC25923 MRSA 64 μg/mL	oxacillin 32-128 μg/mL	[59]
mollicellin W (**46**)	ATCC25923 MRSA 32 μg/mL	oxacillin 32-128 μg/mL	[59]
mollicellin X (**47**)	ATCC 29213 MRSA 64 μg/mL	oxacillin 32-128 μg/mL	[59]
mollicellin Y (**48**)	ATCC29213 MRSA 128 μg/mL<br>ATCC33592 MRSA 128 μg/mL<br>ATCC43300 MRSA 64 μg/mL	oxacillin 32-128 μg/mL	[59]
rhexocerin A (**49**)	T144 MRSA 16 μg/mL	vancomycin 2 μg/mL	[61]
rhexocercosporin A (**51**)	T144 MRSA 32 μg/mL	vancomycin 2 μg/mL	[61]
rhexocercosporin C (**53**)	T144 MRSA 16 μg/mL	vancomycin 2 μg/mL	[61]
rhexocercosporin D (**54**)	T144 MRSA 16 μg/mL	vancomycin 2 μg/mL	[61]
rhexocercosporin E (**55**)	MRSA T144 4 μg/ml	vancomycin 2 μg/mL	[61]
talaromurolide A (**57**)	*S. aureus* 64 μg/mL	chloramphenicol 8 μg/mL	[62]
epicoccether K (**58**)	*S. aureus* 50 μg/mL	cefradine 12.5 μg/mL	[63]
epicoccether L (**59**)	*S. aureus* 50 μg/mL	cefradine 12.5 μg/mL	[63]
epicoccether M (**60**)	*S. aureus* 100 μg/mL	cefradine 12.5 μg/mL	[63]
epicoccether N (**61**)	*S. aureus* 100 μg/mL	cefradine 12.5 μg/mL	[63]
dalesconoside A (**62**)	MRSA 12.5 μg/mL	ciprofloxacin 0.3125 μg/mL	[64]
dalesconoside B (**63**)	MRSA 50 μg/mL	ciprofloxacin 0.3125 μg/mL	[64]
dalesconoside C (**64**)	MRSA 25 μg/mL	ciprofloxacin 0.3125 μg/mL	[64]
dalesconoside D (**65**)	MRSA 50 μg/mL	ciprofloxacin 0.3125 μg/mL	[64]
dalesconoside F (**66**)	MRSA 50 μg/mL	ciprofloxacin 0.3125 μg/mL	[64]
phyligustricin C (**67**)	*S. aureus* 16 μg/mL	chloramphenicol 4 μg/mL	[65]
phyligustricin D (**68**)	*S. aureus* 16 μg/mL	amoxicillin 0.125 μg/mL<br>chloramphenicol 4 μg/mL	[65]
1-(3-hydroxy-1-(hydroxymethyl)-2-methoxy-6-methylnaphthalen-7-yl)propan-2-one (**70**)	MRSA 10.2 ± 1.8 8mm	30 μg of vancomycin 32 mm	[67]
1-(3-hydroxy-1-(hydroxymethyl)-6-methyl-naphthalene-7-yl)propan-2-one (**71**)	MRSA 11.3 ± 2.0 mm	30 μg of vancomycin 32 mm	[67]
stagonosporopsin C (**73**)	subsp. aureus MIC_{50} 41.362 μM	penicillin G sodium salt 1.963 μM	[69]
Lawsozaheer (**75**)	*S. aureus* 84.26% inhibition at 150 μg/mL	ofloxacin 87.013% inhibition at 100 μg/mL	[71]

Table S4 (Cont.).

koninginin W (**76**)	*S. aureus* 256 μg/mL	kanamycin 128 μg/mL<br>ampicillin 8-16 μg/mL	[71]
pestallic acid T (**80**)	*S. aureus* and MRSA 20 μg/mL	N.A.	[74]
atrovinol (**81**)	*S. aureus* 8 μg/mL	N.A.	[75]
penioctadecatrienoic A (**86**)	*S. aureus* 32 μg/mL	N.A.	[77]
epicolidine B (**89**)	*S. aureus* 64 μg/mL<br>MRSA 16 μg/mL	vancomycin 0.5 μg/mL<br>vancomycin 0.25 μg/mL	[79]
epicolidine C (**90**)	*S. aureus* 2 μg/mL<br>MRSA 2 μg/mL	vancomycin 0.5 μg/mL<br>vancomycin 0.25 μg/mL	[79]
perinadine D (**91**)	MRSA 125 μM<br>MSSA 125 μM	ciprofloxacin 0.94 μM<br>ciprofloxacin 0.47 μM	[80]
perinadine E (**92**)	MRSA 62.5 μM<br>MSSA 125 μM	ciprofloxacin 0.94 μM<br>ciprofloxacin 0.47 μM	[80]
xylarchalasin A (**96**)	*S. aureus* 100 μg/mL	ampicillin 3.125 μg/mL	[83]
xylarchalasin B (**97**)	*S. aureus* 50 μg/mL	ampicillin 3.125 μg/mL	[83]
xylariachalasin B (**98**)	MRSA 25 μM<br>*S. aureus* 12.5 μM	ampicillin 0.25 μM	[84]
xylariachalasin C (**99**)	MRSA 50 μM	ampicillin 0.25 μM	[84]
Fusarioxazin (**102**)	*S. aureus* 14.8 ± 0.19 mm	ciprofloxacin 16.9 ± 0.13 mm	[86]
peniazaphilone A (**105**)	MRSA 12.5 μM	ampicillin 6.4 μM	[88]
6-(4-methoxyphenoxy)-4-methoxy-2-me-thyl-1H-indole (**107**)	MRSA 22.4 ± 2.4 mm	vancomycin 32 mm	[89]
6-(3,5-dimethoxy-phenoxy)-4-methoxy-2-methyl-1H-indole (**108**)	MRSA 24.6 ± 2.2 mm	vancomycin 32 mm	[89]
chromenopyridin A (**112**)	*S. aureus* 62.5 μg/mL	chloramphenicol 8.0 μg/mL	[91]
sarocladilactone A (**115**)	*S. aureus* 64 μg/mL	tobramycin 1 μg/mL	[95]
sarocladilactone B (**116**)	*S. aureus* 4 μg/mL	tobramycin 1 μg/mL	[95]
ganodermaside E (**117**)	*S. aureus* 1.7 mM	N.A.	[96]
ganodermaside F (**118**)	*S. aureus* 2.1 mM	N.A.	[96]
ganodermaside G (**119**)	*S. aureus* 0.9 mM	N.A.	[96]
ganodermaside H (**120**)	*S. aureus* 1.6 mM	N.A.	[96]
acremochlorin S (**126**)	*S. aureus* MIC_{90} = 62.5 mM	moxifloxacin 3.125 μM	[100]
eutyscoparin G (**128**)	MRSA 6.25 μg/mL<br>*S. aureus* 6.25 μg/mL	vancomycin 1.25 μg/mL	[102]
xylariacinol A (**129**)	*S. aureus* 32 μg/mL	kanamycin 1 μg/mL	[103]

Table S4 (Cont.).

xylariacinol D (**130**)	*S. aureus* 32 μg/mL	kanamycin 1 μg/mL	[103]
septoreremophilane D (**131**)	MRSA 50 μM	ampicillin sodium 12.5 μM<br>Fosfomycin sodium 25 μM	[104]
punctaporonin T (**132**)	*S. aureus* IC_{50} = 83 μg/mL	N.A.	[105]

Table S5. Antibacterial activity of compounds against other genus strains

compound	Biological activity	Positive control	reference
Acinetobacter baumannii			
arthrinoid G (**121**)	64 µg/mL	Amikacin 2 µg/mL	[97]
***Bacterium paratyphosum* B**			
Pinophol A (**87**)	50 µg/mL	ciprofloxacin 0.78 µg/mL	[78]
Enterobacter areogenes			
(-)-3-carboxypropyl-7-hydroxyphthalide (**37**)	12 µg/mL	ampicillin 0.78 – 3.125 µg/mL	[54]
(-)-3-carboxypropyl-7-hydroxyphthalide methylester (**38**)	12.5 µg/mL	ampicillin 0.78 – 3.125 µg/mL	[54]
Enterococcus faecalis			
dalesconoside A (**62**)	25 µg/mL	Ciprofloxacin 0.625 µg/mL	[64]
dalesconoside C (**64**)	25 µg/mL	Ciprofloxacin 0.625 µg/mL	[64]
dalesconoside D (**65**)	50 µg/mL	Ciprofloxacin 0.625 µg/mL	[64]
sucurchalasins A (**93**)	3.1 µg/mL	ciprofloxacin 1.6 µg/mL	[81]
sucurchalasins B (**94**)	3.1 µg/mL	ciprofloxacin 1.6 µg/mL	[81]
Enterococcus faecium			
epicolidines B (**89**)	64 µg/mL	vancomycin 0.5 µg/mL	[79]
epicolidines C (**90**)	8 µg/mL	vancomycin 0.5 µg/mL	[79]
Erwinia carotovora			
2,6-dimethyl-5-methoxyl-7-hydroxylchro-mone (**74**)	100 µg/mL	streptomycin 50 µg/mL	[70]
***Klebsiella* sp.**			
Penicimenolide G (**42**)	8 mm (1 mg/disc)<br>10 mm (1.5 mg/disc)<br>13 mm (2 mg/disc)	amoxicillin 0.0 mm (1 mg/disc)	[56]
Klebsiella pneumoniae			
arthrinoid G (**121**)	8 µg/mL	Amikacin 2 µg/mL	[97]
Micrococcus lysodeikticus			
(-)-3-carboxypropyl-7-hydroxyphthalide (**37**)	50 µg/mL	ampicillin 0.78 – 3.125 µg/mL	[54]
Mycobacterium tuberculosis			
diaportheremophilin B (**122**)	50 µg/mL	rifampicin 0.00625 µg/mL<br>streptomycin 0.313 µg/mL<br>isoniazid 0.0938 µg/mL<br>ethambutol 0.469 µg/mL	[98]
diaportheremophilin E (**123**)	25 µg/mL	rifampicin 0.00625 µg/mL<br>streptomycin 0.313 µg/mL<br>isoniazid 0.0938 µg/mL<br>ethambutol 0.469 µg/mL	[98]
punctaporonin T (**132**)	IC_{50} = 36.8 µg/mL	N.A.	[105]

Table S5 (Cont.).

Ralstonia solanacearum			
Isotalaroflavone (**34**)	64 μg/mL	streptomycin sulfate 8 μg/mL	[52]
ganodermasides E (**117**)	3.3 mM	N.A.	[96]
ganodermasides F (**118**)	1.0 mM	N.A.	[96]
ganodermasides G (**119**)	0.4-0.9 mM	N.A.	[96]
ganodermasides H (**120**)	0.8-1.6 mM	N.A.	[96]
Salmonella typhimurium			
Talaromurolide A (**57**)	64 μg/mL	chloramphenicol 8 μg/mL	[62]
Koninginin W (**76**)	64 μg/mL	kanamycin 16-32 μg/mL<br>ampicillin 128-256 μg/mL	[72]
xylariachalasin C (**99**)	25 μM	ampicillin 0.25 μM	[84]
Shigella dysenteriae			
(-)-3-carboxypropyl-7-hydroxyphthalide (**37**)	12 μg/mL	ampicillin 0.78 – 3.125 μg/mL	[54]
**vancomycin-resistant *Enterococcus faecalis***			
**compound**	**Biological activity**	**Positive control**	**reference**
subplenone A (**14**)	2 μg/mL	levofloxacin 1 μg/mL	[42]
subplenone E (**15**)	2 μg/mL	levofloxacin 1 μg/mL	[42]
subplenone G (**16**)	4 μg/mL	levofloxacin 1 μg/mL	[42]
Rhexocerin A (**49**)	16 μg/mL	vancomycin >128 μg/mL	[61]
rhexocercosporin A (**51**)	8 μg/mL	vancomycin >128 μg/mL	[61]
rhexocercosporin B (**52**)	8 μg/mL	vancomycin >128 μg/mL	[61]
rhexocercosporin C (**53**)	16 μg/mL	vancomycin >128 μg/mL	[61]
rhexocercosporin D (**54**)	8 μg/mL	vancomycin >128 μg/mL	[61]
rhexocercosporin E (**55**)	4 μg/mL	vancomycin >128 μg/mL	[61]
**Vancomycin resistant-*Enterococcus faecium***			
subplenone A (**14**)	0.5 μg/mL	levofloxacin >64 μg/mL	[42]
subplenone E (**15**)	0.5 μg/mL	levofloxacin >64 μg/mL	[42]
subplenone G (**16**)	1.0 μg/mL	levofloxacin >64 μg/mL	[42]
epicolidines B (**89**)	16 μg/mL	vancomycin >16 μg/mL	[79]
epicolidines C (**90**)	2 μg/mL	vancomycin >16 μg/mL	[79]
***Xanthomonas axonopodis* pv. phaseoli**			
3-methoxy-5-methylnaphthalene-1,7-diol (**69**)	1.25 mg/mL (7.67 ± 0.33 mm)	N.A.	[66]
***Xanthomonas oryzae* pv. oryzicola**			
(±)-trichodermatrione A (**13**)	64 μg/mL	streptomycin sulfate 16 μg/mL	[41]
Isotalaroflavone (**34**)	64 μg/mL	streptomycin sulfate 16 μg/mL	[52]

Table S5 (Cont.).

***Xanthomonas oryzae* pv. oryzae**			
(±)-trichodermatrione A (**13**)	64 μg/mL	streptomycin sulfate 32 μg/mL	[41]
Isotalaroflavone (**34**)	16 μg/mL	streptomycin sulfate 32 μg/mL	[52]
